# A rare desmoid tumor arising from the manubrium

**DOI:** 10.1186/s13019-015-0240-y

**Published:** 2015-03-18

**Authors:** Chenyang Ye, Guofei Zhang, Ying Chai

**Affiliations:** Department of Thoracic Surgery, the Second Affiliated Hospital, College of Medicine, Zhejiang University, No. 88 Jiefang Rd, Hangzhou, 310009 China

**Keywords:** Desmoid tumors, Manubrium, Sternal reconstruction, Autogenous rib grafts

## Abstract

Desmoid tumors are rare soft tissue tumors derived from fascia and connective tissue of the muscular layers. The abdominal region is the most frequent site of involvement, whereas involvement of sternal manubrium is rare. We report the case of a rare desmoid tumor in the sternal manubrium mimicking radiological and metabolic features of malignant tumor, which was successfully treated by sternal resection and reconstruction with autogenous rib grafts.

## Background

Desmoid tumors are rare, benign, soft-tissue neoplasms, and are characterized by infiltrative growth and a tendency towards local recurrence. Common sites of desmoid tumors include abdominal wall, extremities, shoulder, neck, and chest wall. The sternum is the least common site. We herein report a rare desmoid tumor of sternal manubrium mimicking malignant tumor such as chondrosarcoma, which was successfully treated by complete resection of the tumor and immediate sternal reconstruction with autogenous rib grafts.

## Case presentation

**Figure 1:**
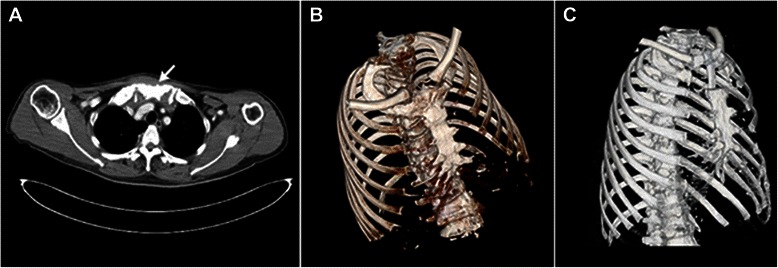
**Radiologic images.** Chest CT axial view **(A)** and computed tomographic scan of the reconstructed chest **(B)** before surgery showed the osteolytic lesion (arrow) in the manubrium. Computed tomographic scan of the reconstructed chest **(C)** was followed up 3 months postoperatively.

**Figure 2:**
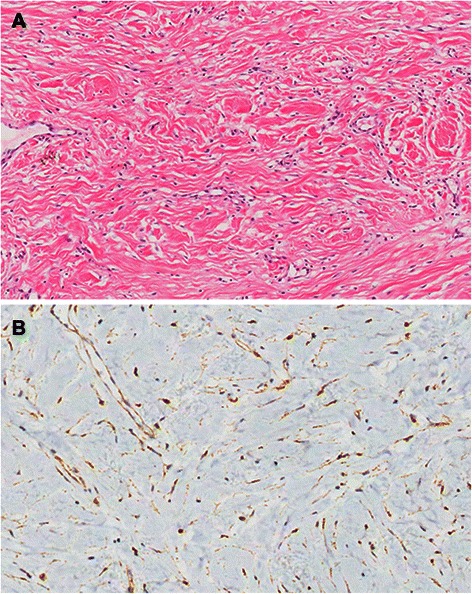
**Histologic findings after surgery.** Histopathological picture **(A)** of desmoid tumor showed a tumor consisting of spindle-shaped cells and collagen fibers (HE × 200). **(B)** Immunoreactivity for beta-catenin was seen within nuclei of tumor cells. (IHC × 200).

A 64-year-old man was referred to our hospital with bulging of the sternal manubrium. He was otherwise asymptomatic. There was no antecedent history of Gardner syndrome, trauma or surgery. Physical examination revealed a firm, 3× 3 cm palpable sternal mass. The contrasted computed tomography (CT) scan of the chest showed a 4× 4 cm mass based on the sternal manubrium, with no apparent involvement of the mediastinal structures (Figure 1A and B). ^18^F-fluorodeoxyglucose–positron emission tomography (FDG-PET) revealed metabolically active areas around the manubrium, both sternoclavicular joints, and bilateral first sternocostal joints, which was suggestive of malignant tumor of the sternal manubrium. The patient refused to undergo preoperative fine-needle aspiration of the lesion. Thus the patient underwent a radical en bloc resection of the tumor and sternal reconstruction with autogenous rib grafts. At surgery, an expansile lesion involving the entire manubrium, both sternoclavicular joints, and bilateral first sternocostal joints was found. En bloc radical manubriectomy was performed including portions of the bilateral clavicles and first ribs. The specimen was removed 4 cm beyond the margin of the lesion. Then through a left anterior lateral incision, about 10 cm of the left fifth rib was harvested from the subperiosteum and carved into two parts with preservation of intact periosteum and pleura. Slots were cut into the ends of the two rib clips, bilateral clavicles, and the edge of the sternum using an electric drill. The two rib grafts were placed to form a T shape between the edges of the residual sternum and bilateral clavicles using rib nails, and then fixed with stainless steel wires. Histopathological examination revealed long fascicles of spindle-shaped cells with slight atypia arranged in a collagenous stroma, indicating a diagnosis of desmoid tumor (Figure 2A and B). The resection margins did not show any signs of neoplastic infiltration. Postoperative recovery was unremarkable and there were no complications. Three months postoperatively, computed tomography scan of the reconstructed chest showed neither instability of the chest wall nor evidence of tumor recurrence (Figure 1C). The patient has been free of disease for 46 months after surgery without any other treatment.

## Discussion

Desmoid tumors are relatively rare, benign, soft-tissue neoplasms most often derived from fascial or musculoaponeurotic structures, accounting for 0.03% of all neoplasms [1]. This tumor has a strong tendency to arise in the abdomen or the chest wall, whereas involvement of the sternal manubrium is extremely rare. The chief complaint is often the presence of a firm, painless mass fixed to the underlying structures. Although the etiology has not been well defined, there are some recognized factors of risk, such as a history of trauma or surgery, familial adenomatous polyposis, Gardner syndrome and estrogen [1]. However, the cause in our case was not clear because the patient was an elderly man who had no antecedent history of Gardner syndrome, trauma or surgery.

CT and FDG-PET can reveal the size, location and metabolic feature of tumors, but histopathological assessment is required to make the definitive diagnoses when the main differential diagnoses are malignant tumors such as chondrosarcoma, as in our patient. Preoperative biopsies or surgical removals are often performed to obtain the final diagnoses. Our patient chose surgical resection of the lesion directly without preoperative biopsy. Histopathologically, the desmoid tumors are mainly characterized by focal increased cellularity, rare mitotic activity, bland cytology, and absence of herringbone pattern, which are different from chondrosarcoma [2].

Desmoid tumors are locally aggressive tumors with a high local recurrence rate [3]. If medically and technically feasible, wide radical resection of the tumors with negative microscopic margins is the best treatment option. Regular chest CT scan needs to be closely followed. In this report, radical en bloc resection with a clear resection margin was performed, and the patient was still well without recurrence at 46 months of clinical follow-up.

The autogenous rib has been considered as one of the most efficacious materials to be used in sternal reconstruction. In our initial report, we proposed the use of autogenous rib grafts as a safe and effective choice for the reconstruction of sternal defects [4]. In this case, our patient successfully underwent surgical resection of the desmoid tumor and immediate sternal reconstruction with autogenous rib grafts. No other treatment was administered. The patient was in good clinical condition at 46 months of follow-up.

In conclusion, we describe an extremely rare case of desmoid tumor in the sternal manubrium mimicking malignant tumor, which was successfully treated by complete resection of the tumor and immediate sternal reconstruction with autogenous rib grafts. The patient has been disease free after 46 months of follow up without any other treatment. We consider that the sternal reconstruction with autogenous rib grafts is a simple and effective solution to a complex problem.

## Conclusions

Although desmoid tumors of the sternum have been described in a few case reports, they are still rare entities. Radical en bloc resection of desmoid tumor of the sternum with a negative margin and sternal reconstruction with autogenous rib grafts is safe, effective and feasible.

## Consent

Written informed consent was obtained from the patient for publication of this Case report and any accompanying images. A copy of the written consent is available for review by the Editor-in-Chief of this journal.

## Abbreviations

CT: Computer tomography
FDG-PET: ^18^F-fluorodeoxyglucose–positron emission tomography
